# Influences of Flood Conditions on Dynamic Characteristics of Novel 3D-Printed Porous Bridge Bearings

**DOI:** 10.3390/ma16062288

**Published:** 2023-03-13

**Authors:** Pasakorn Sengsri, Sakdirat Kaewunruen

**Affiliations:** Laboratory for Track Engineering and Operations for Future Uncertainties (TOFU Lab), School of Engineering, The University of Birmingham, Edgbaston, Birmingham B15 2TT, UK

**Keywords:** novel 3D-printed porous bridge bearings (3DPPBBs), dynamic modal parameters, idealised single degree of freedom (ISDOF), flood, climate change adaptation, structural dynamics

## Abstract

As the key safety-critical component of a bridge support system, bridge bearings are extensively used to accommodate, balance, and transfer differential displacements and loads between the superstructure and substructure of a bridge during operations. Several studies have been conducted to obtain dynamic modal parameters of traditional bridge bearings only in perfectly dry environments. However, in extreme weather conditions (e.g., heavy rain, flash floods, etc.), water can ingress and change the bearings’ properties. In this study, novel 3D-printed porous bridge bearings (3DPPBBs) have been fabricated by Fused Deposition Modeling (FDM) with thermoplastic polyurethane (TPU) filaments. This study is the first to determine the influences of flood conditions on their dynamic properties, which has never been done before. An idealised single degree of freedom (ISDOF) for these novel bearings is considered for the non-destructive field-testing technique of the critical bridge component. A series of experimental tests have been performed under several conditions of flooding levels. The new results unprecedentedly indicate that relatively higher dynamic damping ratios can be found with the increasing flood levels. In contrast, the natural frequencies and dynamic stiffness decrease with the same conditions. Novel insights are essential for bridge engineers to assess and monitor bridge vibrations exposed to extreme weather conditions.

## 1. Introduction

Bridge bearings play an important role in a bridge system. Their function is to transfer/facilitate loads/displacements between the superstructure and substructure of a bridge, when the bearings are subject to either static loading or dynamic loading during operation. Another function of bearings is to support the weight of the bridge superstructure. Typical bridge bearings well-known as elastomeric ones are a combination of elastomeric layers and with/without any reinforcement. For example, bridge bearings without reinforcement referred to as plain elastomeric bridge bearings (PEBBs) are required for tiny bridge systems experiencing small horizontal displacements, whilst bridge bearings with reinforcement called steel/fibre-reinforced elastomeric bridge bearings (S/FREBBs) are used to address high horizontal and rotational displacements for relatively large bridges.

In our critical review [1], during dynamic loads, bridge bearings can experience a sudden phenomenon called resonance, which often occurs when the bridge system has its natural frequency equal to the driving frequency from live loads. This leads to the failure of the bridge under vibration. In terms of their static behaviour [2,3,4,5], bridge bearings can fail in compression due to the bulging behaviour of rubber and also can face stress concentrations of the interaction between steel plates and rubber layers. Additionally, they are vulnerable to tension loading (uplift), resulting in local failures in the inner structure of rubber called a cavitation phenomenon when the bearings are connected to top and bottom plates with bolted connections [6,7,8,9]. The development of these common bearings used for eliminating these problems are considered to replace steel plates and rubber layers with alternative materials and porous structures due to their heavy weight and high labour costs [10,11]. With the help of a porous structure’s performance [12,13,14,15,16,17], bridge bearings, which have a relatively high performance to weight ratio, could offer better properties under various loading conditions, especially for layer-by-layer deformation behaviour and vibration attenuation (increase in a fundamental natural period of a structure) under compression and vibration, respectively.

As mentioned previously, the failures and inadequate structure designs of these typical bridge bearings under extremely various loading [18,19], especially for compression and vibration loading, can be prevented better by using an extremely lightweight structure with a 3D printing material possessing rubber-like properties [20,21]. At this point, the development of common elastomeric bridge bearings to eliminate the drawbacks of the common bridge bearings has inspired us to design novel porous bridge bearings having relatively high performance to weight ratios manufactured by additive manufacturing approach and to investigate its behaviour imposed to static and dynamic loading. From our previous works [22,23,24,25], Sengsri and Sakdirat have studied the local buckling behaviour of a meta-functional auxetic (MFA) composite bridge bearing model using an auxetic sandwich core generated from 75 single auxetic unit cells under compression. It is found that the local buckling failures of MFA unit cells can possibly sustain before yielding under compressive load due to their local slenderness ratio. Nevertheless, this bearing model subjected to a compression force exhibits an auxetic behaviour with high structural crashworthiness for bridge bearing applications.

Another research study from the authors has been conducted on the identification of mechanical properties and energy-absorption capability of a 3D-printed triply periodic minimal surface (TPMS) sandwich lattice bridge bearing model without a thickness under combined compression-shear loading. The results have clearly shown that the proposed lattice model with at least six-unit cells under the combined compression-shear load can reach the mechanical responses and specific energy absorption of an elastomeric block unit cell. Moreover, the shear force and the specific energy absorption of the lattice model are relatively higher under combined compression-shear loading and uniaxial compression loading, respectively. Its failure modes for bridge bearing application observed through the stress-stain curve of the lattice model under both the same conditions are identified as elastic-plastic and hysteretic behaviours. It is evident that there are three main factors (material, structure geometry, and volume fraction) which dominate these two behaviours of the TPMS lattice model [26].

Since the benefits of using these developed structures for bridge bearing applications, they are likely to be used in bearings’ structure under any seismic action, potentially offering better physical and mechanical properties, including crashworthiness, better energy absorption, vibration attenuation, lightweight, eco-friendly and recycling materials, and so on. According to the behaviours of these developed bridge bearings have been investigated under static loading with only dry conditions. It is necessary to understand their behaviour affected by floods, which has never been investigated. The effect of floods on railway structure could result in the change of dynamic material properties [27]. To investigate the vibration behaviour of porous bridge bearings under flood conditions, modal testing and analysis, a suitable tool used to obtain the dynamic parameters of a structure under flood conditions is considered in this paper [28,29]. 

To best of our knowledge following a critical review of the available literature, there are no current works focusing on the influence of the flood conditions on the dynamic modal paraments of 3D-printed porous bridge bearing (3DPPBBs) using gyroid unit cells under vibration with various flood conditions. In this paper, we aim to determine the dynamic modal paraments of the 3DPPBBs affected by flood conditions, and also to develop a numerically vibrating model from these dynamic modal parameters for practical use under various flood conditions. Moreover, this model can potentially be a promising alternative for 3D-printed porous bridge bearings with a high-performance to density ratio under bridge operations with severe environmental conditions. Materials and methods, including the process of manufacturing specimens of this gyroid structure, will be detailed in the following section. The impact hammer excitation method is used in the paper due to the ease of setup and the possibility of complete tests, when considering the size and weight of specimens as well as their range of frequency excitation. Subsequently, the experimental measurements and the discussion of the experimental and numerical analysis will be presented. Finally, the proposed gyroid bridge bearing model subjected to vibration with various flood conditions is generated using the dynamic modal parameters from the experiment data, in order to predict its behaviour in practical use.

## 2. Novel 3D Printed Porous Bridge Bearings (3DPPBBs)

### 2.1. Design of a Novel Porous Bridge Bearing Model

**Figure 1:**
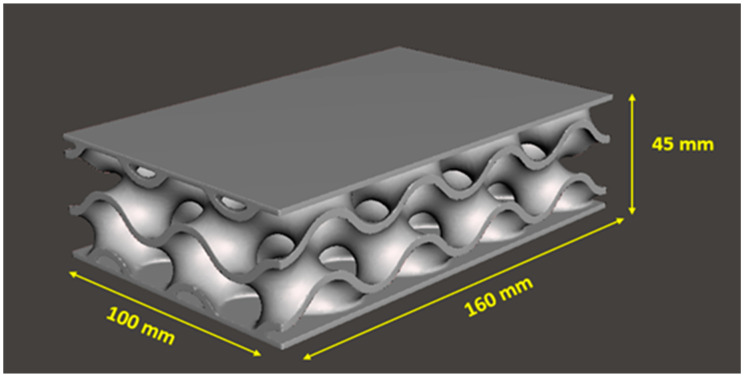
Illustrating the gyroid bridge bearing model from CAD software.

Figure 1 presents the designed 3D porous bridge bearing model using several gyroid cellular structures. Gyroid surface is one of the most used triply periodic minimal surfaces for many applications, which offers a relatively high performance to weight ratio under any loading, especially for stiffness. There are numerous ways to generate a structure model such as parametric generation based on atomic units and implicit surface extraction construction. In this paper, the parametric generation approach has been selected for creating the proposed model using CAD software due to its easy modification, instant design as well as better resolution. The model dimensions are based on the design of common bridge bearings used for Thailand highway bridges [30]. The model consists of two layers with a 50 mm unit cell size and a 2 mm thickness. The gyroid geometry is based on the following equation.

(1) $$sin\left(\frac{2\pi x}{l}\right)*cos\left(\frac{2\pi y}{l}\right)+sin\left(\frac{2\pi y}{l}\right)*cos\left(\frac{2\pi z}{l}\right)+sin\left(\frac{2\pi z}{l}\right)*cos\left(\frac{2\pi x}{l}\right)=t,$$

where l denotes the size of a fundamental cell and t determines the cross-section of the sheet, which affects the relative density of the structure. After that, the model CAD file is exported as a STL file for additive manufacturing.

### 2.2. Materials

**Figure 2:**
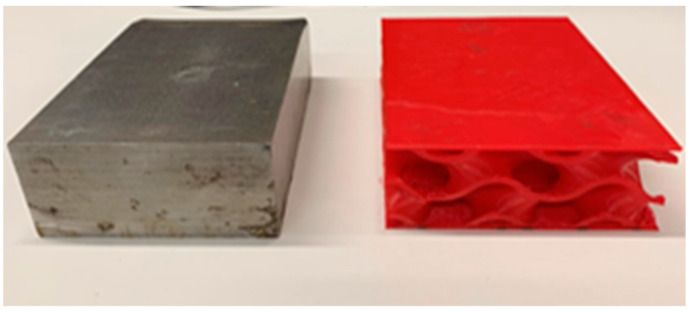
Thick steel plate specimen and the 3D-printed TPU gyroid specimen.

**Figure 3:**
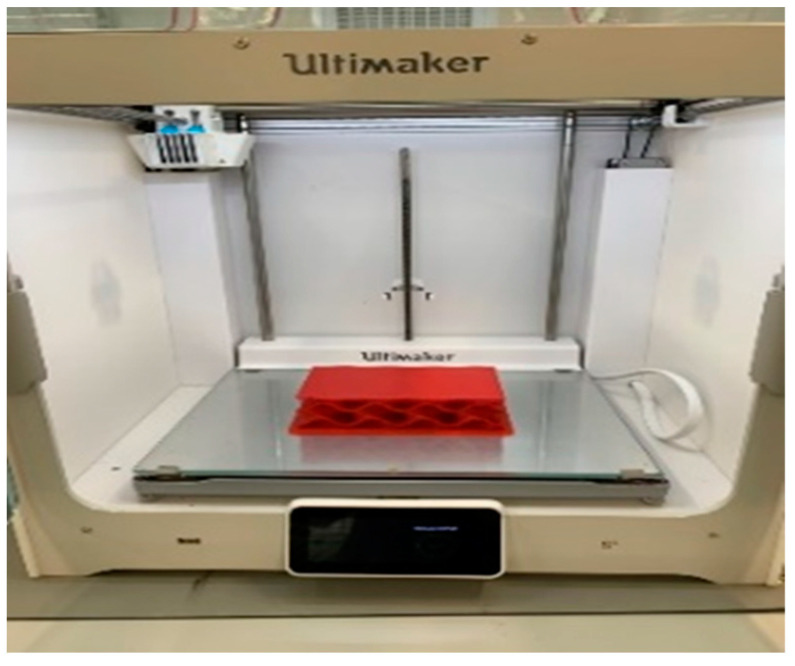
Additive manufacturing for the 3DPPBB specimen using an Ultimaker 3D printer.

The specimen materials are stainless steel and thermoplastic polyurethane (TPU) considered as the mass and spring, respectively (indicated in Figure 2). The fused deposition modeling (FDM) 3D Printing technique is selected for producing the bearing specimens, which needs the features of a University of Birmingham’s Ultimaker 3D printer, shown in Figure 3. The TPU material is chosen due to its rubber-like properties [31,32,33,34] and manufacturing ability in the market. Table 1 provides the physical and mechanical properties of stainless steel and thermoplastic polyurethane (TPU) considered as the mass and spring.

### 2.3. The Preparation of the 3D-Printed Porous Bridge Bearing under Flood Conditions

**Figure 4:**
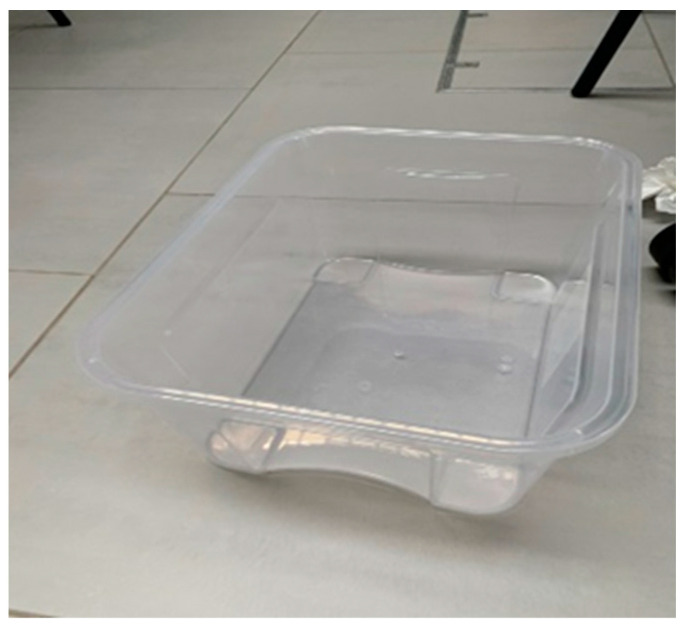
Showing a clear plastic box for flood conditions.

For different flood conditions, a small container which is a transparent plastic box (Figure 4) with a size of 45 × 40 × 15 cm is used for tests in this paper. The side mark is utilised to adjust the flooding level every 25 per cent.

## 3. Methods

**Figure 5:**
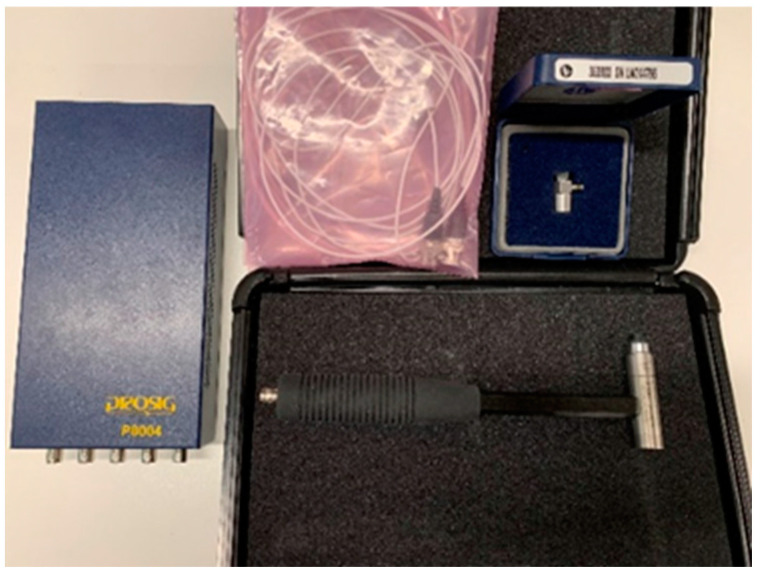
Impact hammer, accelerometer, and the acquisition device with DATs software.

**Figure 6:**
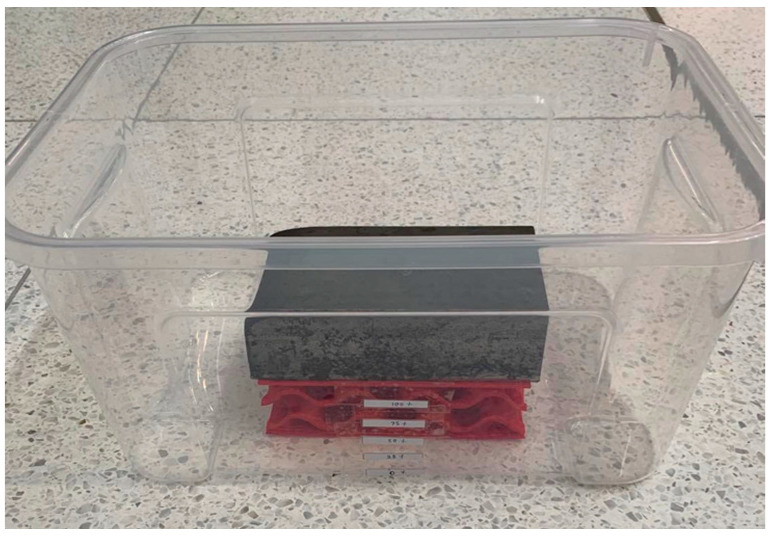
Demonstrating the preparation of test setup for dry and flood conditions.

**Figure 7:**
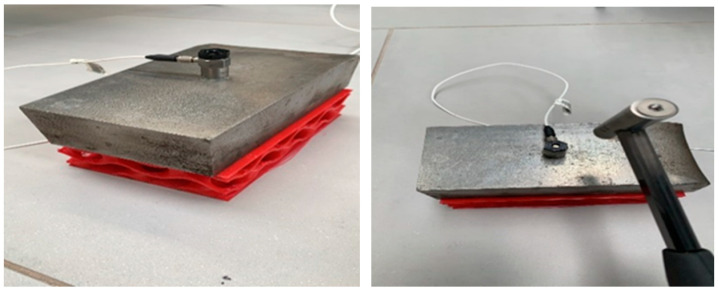
Test setup of the single mass-spring 3DPPBB system used to verify the experiments.

The modal testing is considered to conduct the experiments with help of a Prosig system (DATs) [35]. In terms of data collection, Figure 5 shows the instrumented hammer and acquisition device used to excite a vibration force and convert signals and then record the data, respectively. After that, modal parameters are identified using datafit curve fitting software. The flood level is basically measured and collected as illustrated in Figure 6. Finally, the experimental verification compared to numerical predictions in Abaqus is performed to make sure that the modal testing is valid with the assumptions as described in the latter section. It is important to mention that the test setup illustrated in Figure 6 and Figure 7 is considered as a single degree of freedom (SDOF) system.

In this study, we aim to determine the dynamic modal parameter of a single-degree-of-freedom mass-spring system supported by a novel TPMS bridge bearing under various flood conditions. Furthermore, the proposed model is established and developed by using these modal parameters. Impact hammer testing is a well-known non-destructive approach based on instrumented hammer impact excitation and a signal-processing analysis to measure the vibration response of the structure to impact excitation. In order to obtain the frequency response function (FRF), the Fourier transformation is used to convert the vibration signals to the FRF. Then, it is employed to extract the dynamic modal parameters and the dynamic properties [36].

### 3.1. Determination of Dynamic Modal Parameters of the 3DPPBB System Using an Impact Hammer

**Figure 8:**
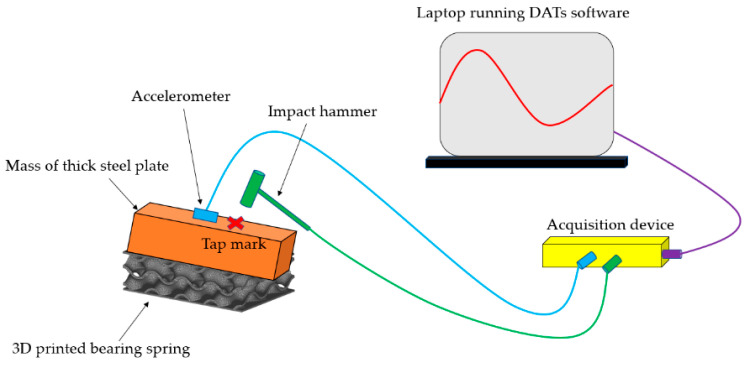
Record data employing the tap hammer and the acquisition device.

An impact hammer force (tap force) and accelerometer records are proper equipment for modal analysis, if the dynamic properties are found in a range between middle and high frequency domains. Figure 8 shows the process of capturing data for modal testing using the instrumented hammer. The fast Fourier transform (FFT) is utilised to convert a range of time recordings into an average Frequency Response Function (FRF), which corresponds directly to the material property. In other words, a FRF is a function employed to measure the response of a system to an excitation, divided by the excitation magnitude, in the frequency domain. Addtionally, the FRF based on monitoring data is basically influenced by the data noise [30].

The record data for analyses in a short time is performed using an acquisition device connected to a laptop. It is important to mention that the idealised concept for lightweight TPMS bridge bearing testing is considered as a lumped mass model (single degree of freedom, SDOF). This can potentially enable the test setup to obtain the direct vicinity of the resonant peak. Regarding the SDOF idealisation of the system, the use of an accelerometer is conducted to measure the response at the middle point with at least 3 hit times and then a range of FRFs obtained are extracted to acquire the natural frequencies and the mode shapes using modal analysis software (DATs).

### 3.2. Simulating Floods in Novel Porous Bridge Bearing

**Figure 9:**
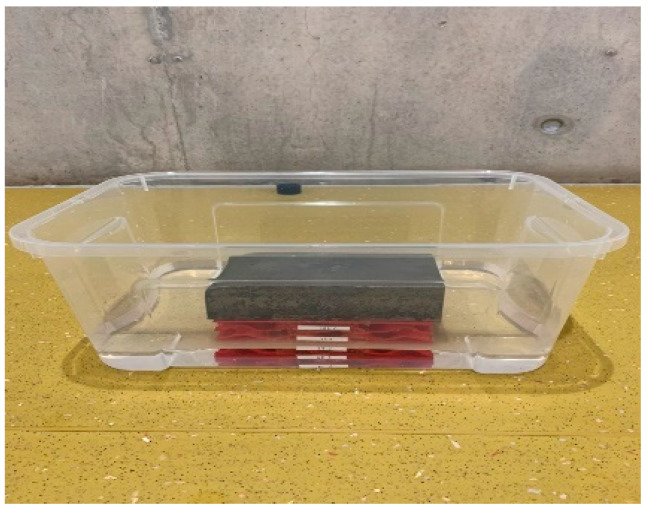
Porous bridge bearing at a flood level.

The flood simulation is presented to consider the effect of flood on the dynamic modal parameters of the lumped mass using the proposed TPMS bridge bearing as a spring on various flood conditions. The dynamic behaviours are measured at every level (increase every 25 per cent from 0 to 100 per cent), as shown in Figure 9. It is important to note that this flood condition is considered as the static flood situation, whereas for the actual flood which can normally flow and have impact effect. This impact effect is not considered in this paper, because the flood flow will affect the vibration of the single degree of freedom bearing system, since the system has an interaction bewteen the lump mass and the bearing as a spring. This might lead to difficulties in obtaining the results of the tests.

### 3.3. Single-Degree-of-Freedom (SDOF) System

**Figure 10:**
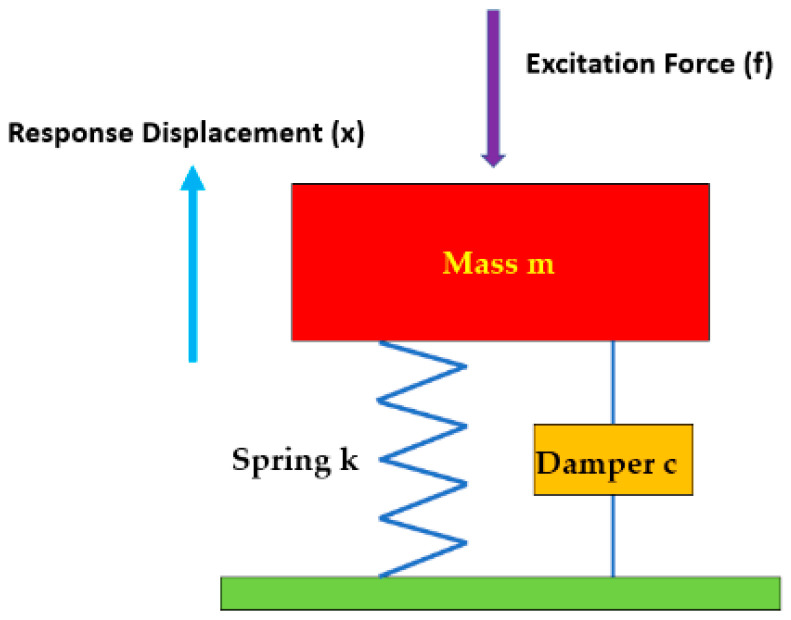
Demonstrating a SDOF discrete parameter model.

In reality, most of physical structures are continuous, their behaviour can generally be represented by a discrete parameter model as illustrated in Figure 10. The idealised components are well-known mass, a damper, a spring, and an excitation force. The first three components describe the physical system. The mass in the system stores kinetic energy and the spring stores elastic potential energy. When the system is excited by a force, this energy enters the system via the force and then it is dissipated through damping. It is worth to mention that in this study the test setup for the proposed TPMS bridge bearing is considered as a SDOF system, hence the multi-degree of freedom system (MDOF) is outside the scope of this study.

In addition to the SDOF system, it is fundamental for the investigation of MDOF systems, as numerous vibration systems employed in today’s engineering are able to be simplified to an SDOF idealisation. Thus, idealised outcomes can be acquired using this theory, and the structural SDOF system can be represented as a single input-single output system [37,38,39], which is commonly applicable to complex engineering structures [40,41].

The idealised components of the physical system can be described by the formula of motion presented in Equation (2).

(2) $$m\ddot{x}\left(t\right)+c\dot{x}\left(t\right)+kx\left(t\right)=f\left(t\right),$$

The solution of a vibration system, $x\left(t\right)$ is assumed to be considered as a form of the following equation.

(3) $$x\left(t\right)=Asin\left(\omega_{n}t+\emptyset \right),$$

This selection is made because the sine function describes oscillation. Where the constant A is the amplitude (maximum value) of the displacement; the angular natural frequency (ω) denotes the interval in time over which the function repeats itself; and ∅ denotes the initial value of the sine function. Equation (4) shows complete differentiation of the displacement.

(4) $$\dot{x}\left(t\right)=\omega_{n}A\cos\left(\omega_{n}t+\emptyset \right),$$

and the acceleration, $\ddot{x}(t)$, expressed by

(5) $$\ddot{x}\left(t\right)=-\omega_{n}^{2}A\sin\left(\omega_{n}t+\emptyset \right),$$

Substitution of Equations (3)–(5) into 2 without damping and excitation force yields

$$-m\omega_{n}^{2}A\sin\left(\omega_{n}t+\emptyset \right)=-kA\sin\left(\omega_{n}t+\emptyset \right),$$

Dividing by m and A yields the fact that this last equation is satisfied if

(6) $$\omega_{n}=\sqrt{\frac{k}{m}},$$

where the value of $\omega_{n}$ characterises the spring-mass system that the frequency at which then motion automatically oscillates without the action of any external driving force and damping force, and thus is called the system’s natural frequency. However, if the system has damping, the Equation (2) becomes

(7) $$\ddot{x}\left(t\right)+2\delta \omega_{n}\dot{x}(t)+\omega_{n}^{2}x(t)=f(t),\;x(0)=x_{0}\;\mathrm{and}\;\dot{x}\left(0\right)=v_{0}$$

In case of an underdamped system (0 < δ<1) with free response, the solution of displacement becomes

$$x(t)=\frac{\sqrt{{\left(v_{0}+\delta \omega_{n}x_{0}\right)}^{2}+{\left(x_{0}\omega_{d}\right)}^{2}}}{\omega_{n}\sqrt{1-\delta^{2}}}e^{-\delta \omega_{n}t}\sin\left(\omega_{d}t+\emptyset \right),$$

where

(8) $$\omega_{d}=\omega_{n}\sqrt{1-\delta^{2}}\;\mathrm{and}\;\emptyset =\tan^{-1}\frac{x_{0}\omega_{d}}{v_{0}+\delta \omega_{n}x_{0}}$$

Here $\omega_{d}$ defines the damped natural frequency and δ determines the damping ratio of the motion system, δ = $\frac{c}{2m\omega_{n}}$.

### 3.4. Impulse Response Function

As aforementioned, the forced response of a single-degree-of-freedom system is considered the particular case of a harmonic driving force, which is sinusoidal of an individual frequency. In this section, the response of a system to an impulse force is investigated. The principle of superposition can be used to determine the response to different combinations of forces dependent on the single response to a particular force, if the response of the system investigated is linear. It is important to note that for the experiments in this paper the transient force only is considered, and the rest of forces are outside the scope of this study.

**Figure 11:**
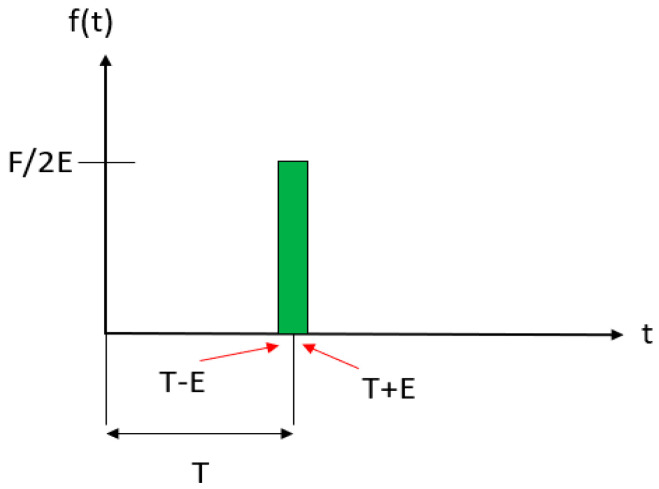
Time history of an impact load used to model impact loading comprising a large magnitude applied for a short time interval.

An impulse force, also well-known as a shock force resulting from a sudden vibration, is a force which is applied for an extremely short time. The impulse is a nonperiodic force. As the aforementioned free response of the system to certain initial conditions, the response of a system to an impact force is the same. This is helpful for many cases when the applied load is impulsive in nature, for example, acts with large magnitude for an extremely short period of time (Figure 11).

Where E is a small positive number. This simple rule, f(t), can be integrated to determine the impulse. The impulse of the force f(t) is determined by the integral, expressed by

(9) $$i\left(E\right)=\int_{T-E}^{T+E}f\left(t\right)dt,$$

Recalling the response of an underdamped single-degree-of-freedom system with zero initial displacement $x_{0}$ = 0 is just

(10) $$x\left(t\right)=\frac{v_{0}}{\omega_{d}}e^{-\delta \omega_{n}t}\sin\omega_{d}t,$$

Substitution of $v_{0}$ = $\hat{F}/m$ = F∆t/m (due to the change in momentum at impact, whilst the initial displacement) into this last expression yields

(11) $$x\left(t\right)=\frac{\hat{F}}{{m\omega }_{d}}e^{-\delta \omega_{n}t}\sin\omega_{d}t,$$

## 4. Results

### 4.1. Experimental Measurements

**Figure 12:**
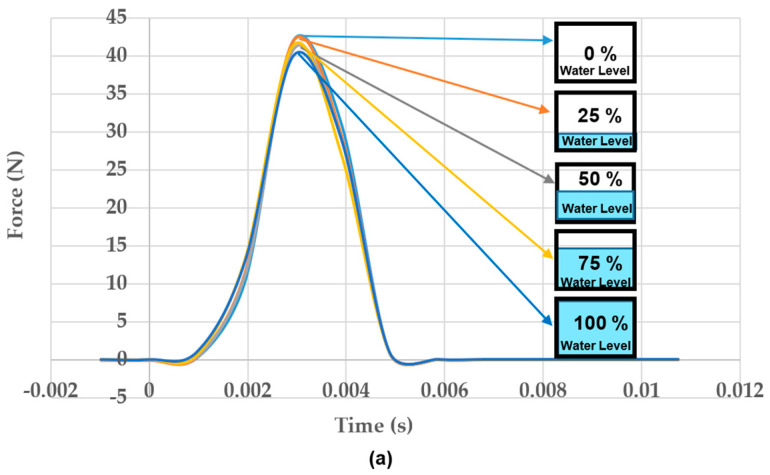

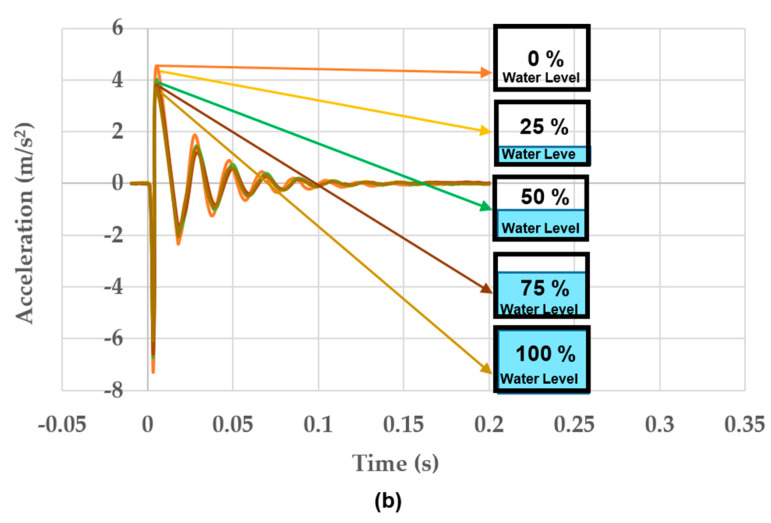
(**a**). Change of impulse, (**b**) change of acceleration under various water levels.

**Figure 13:**
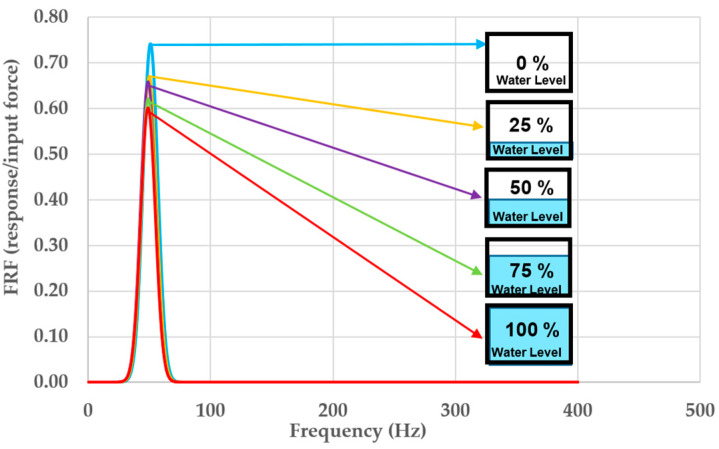
Chart of frequency response function (FRF).

The experimental measurements derived from the modal testing of a thick steel plate over the proposed 3D-printed porous TPU bridge bearing are indicated in Figure 12 and Figure 13. Note that the most possible method to test the proposed 3D-printed TPU bearing is that of using the hammer excitation in vertical direction perpendicular to its top surface, due to the complex geometry and very lightweight. Furthermore, the main load that bearing basically support the superstructure weight of a bridge is applied in that direction before continuing resisting horizonal loads or others.

### 4.2. The Impulse Forces

Figure 12a illustrates the impulse forces within 0.005 s and the amplitudes of these forces occur around at the same time.

### 4.3. The Change of Acceleration

With the increasing water level, the acceleration reduces as shown in Figure 12b. This means that the effect of flood conditions on the dynamic modal parameters of the proposed 3D-printed porous bridge bearing is significant, resulting in the increase in damping of the vibration system. Then, the vibration response of the system is weakened, and the amplitude becomes lower. As such, the entire energy consumption of the proposed porous bridge bearing system is proportional to the flood level.

### 4.4. The Change of Frequency Response Function (FRF)

It is obvious that the FRFs are affected by the water level as seen in Figure 13. The trend of the FRFs is decreasing with increasing the flood level. When the flood level is completely full, its natural frequency significantly changed compared to that of the initial state. Referring to Equation (6), if the natural frequency changes, the stiffness also alters, as the mass is assumed to be constant. It is important to note that this paper only focuses on the peaks of natural frequencies of the proposed bearing’s vibration system rather than considering the noise. 

### 4.5. The Change of Natural Frequency

**Figure 14:**
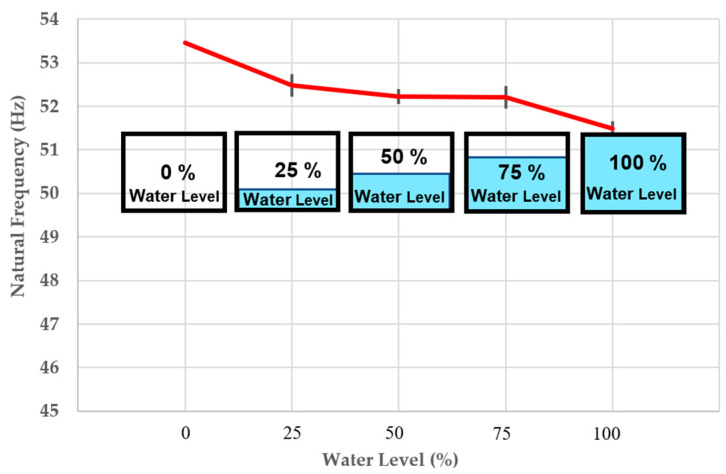
The change of natural frequency.

Figure 14 shows the change of the 3D-printed porous bridge bearing system’s natural frequencies, indicating that there is a dramatic change (a considerable jump, 3.7% lower) after full flood level, but the rest of frequencies of the vibration systems with increasing water level from 0% to 100% (every 25%) is almost unchanged. The reason causing the significant jump is that the lower surface of the steel plate’s mass was likely to come into contact with flood water, resulting in the unusual change of the system’s natural frequency. The overall trend of natural frequency change is decreasing with the increasing water level. Note that, all the three following curves (natural frequency, stiffness, and damping) providing average values are also plotted to compare with standard deviation (gray dash lines in y-axial direction).

### 4.6. The Change of Dynamic Stiffness

**Figure 15:**
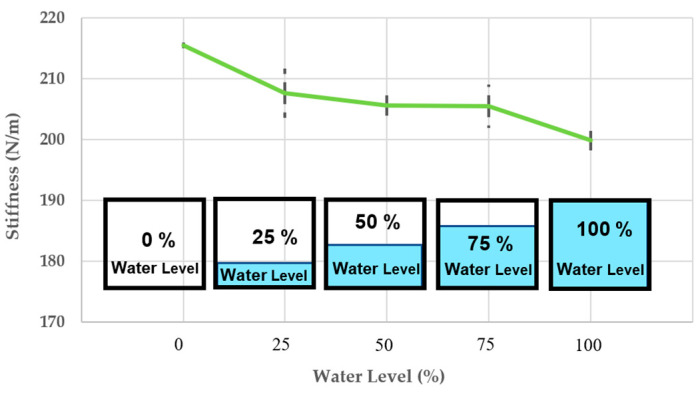
The change of stiffness.

As such the natural frequency decreases when the flood level increases, and the stiffness also decreases as presented in Figure 15. As previously mentioned, the trend of the system’s stiffness is identical to that of natural frequency, but the different values of the modal parameter. This is because the stiffness of the vibration system is proportional to the natural frequency according to Equation (6), and the mass does not affect the relationship between the stiffness and natural frequency of system. The average stiffness continues to lower, compared to the dry condition, and is also nearly 7.5% less than that under dry condition.

### 4.7. The Change of Damping Ratio

**Figure 16:**
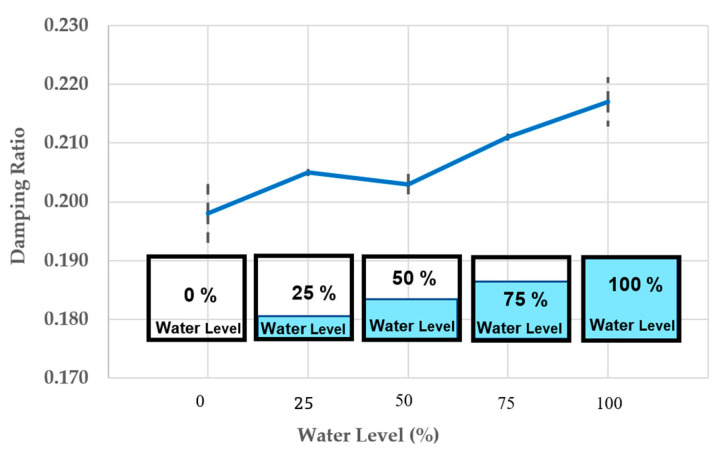
The change of damping.

In short, as presented in Figure 16, the trend of the vibration system’s average dynamic damping continues to rise with increasing the water level. Compared to full flood condition, the average damping value of the 3D-printed bearing material in initial state is approximately 10% lower. The rest under the other conditions is somewhat stable at 4–6% more than that in the initial state. It is important to note that these damping values might be affected by the complexity of the gyroid structure having several holes that the water can enter and change its dynamic modal parameters.

### 4.8. Finite Element Modelling of the Proposed Bridge Bearing

**Figure 17:**
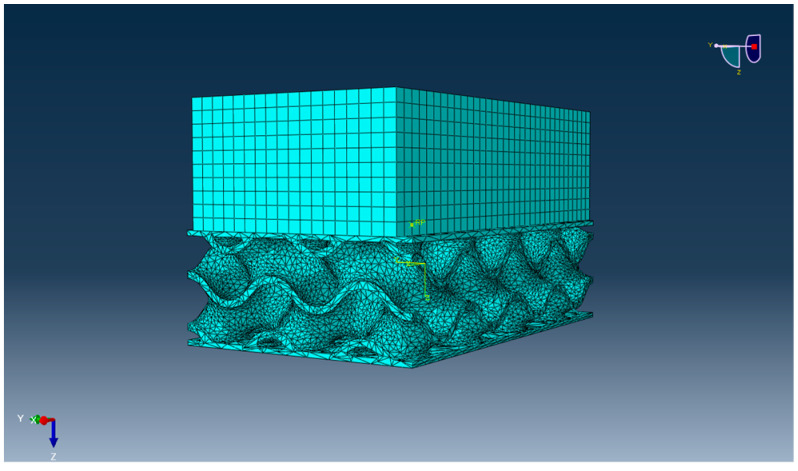
Finite element model setup of the modal analysis in Abaqus software.

According to the experimental results, in this section the development of a 3D-printed gyroid bridge bearing has been proposed and validated against the experimental data in the following section. Figure 17 shows the whole finite element (FA) model consisting of the proposed bearing model and the thick steel plate model considered as a lump mass. The dimensions of both the plate and bearing models are the same as the specimen. Moreover, their material model properties are provided in Table 1. Regarding the mesh generation of both the models, the proposed bearing and the thick steel plate models are discretised with 3D mesh composed of 173,457 ten-node first-order tetrahedral solid elements (C3D10) and 6120 eight-node reduced integration first-order brick elements (C3D8R), respectively. As the consideration of the free vibration of an undamped SDOF system of the proposed bearing on a ground, the lowest mode corresponding to the vertical translation is the same manner in the tests, occuring at a natural frequency of 65 Hz. Alternatively, Equation (6) in Section 3.3 is used to calculate its natural frequency, involving the static parameters such as stiffness and mass. This is becasuse the dynamic model of the proposed bearing requires the static stiffness of material at frequency equal to zero. It is also worth noting that the stiffness of the bearing specimen is assumed within its elastic range. Subsequently, a compariosn of the natural frequencies between the predictions and measurements is conducted in the following section. For the cabrilation of the model under flood conditions, the model will be based on the variations in the damping ratio corresponding to the flood levels, which are obtained from the measurements.

### 4.9. Experimental Justification

**Figure 18:**
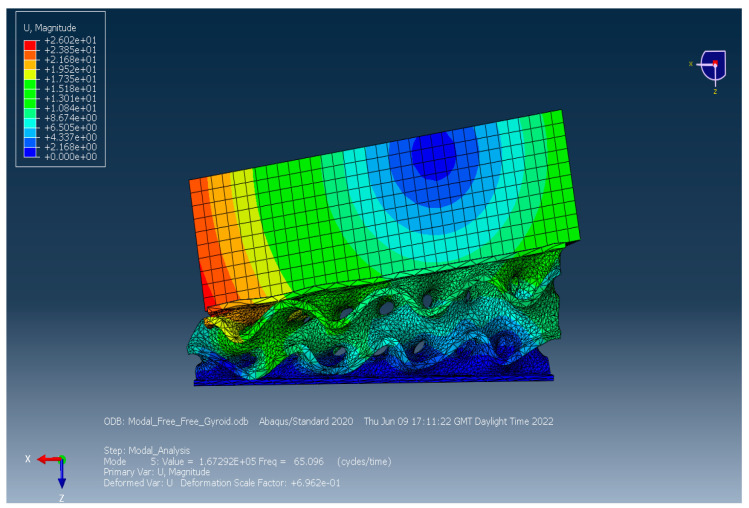
FEA simulation of the proposed 3D-printed gyroid bridge bearing model subjected to free vibration as considered in SDOF.

**Figure 19:**
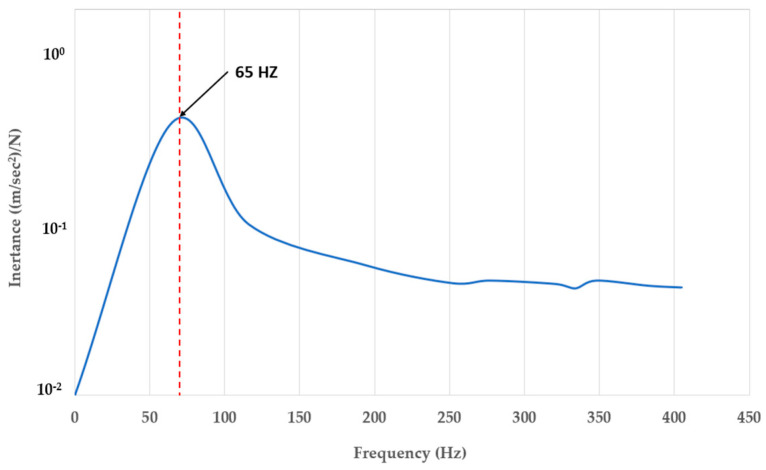
Fundamental resonance frequency of SDOF of the bearing system resulting from the modal testing.

The numerical prediction resulting from the modal analysis of a thick steel plate over the proposed 3D-printed porous TPU bridge bearing under dry condition is conducted in Abaqus software, as indicated in Figure 18 and Figure 19 (plotted as log amplitude versus frequency). Moreover, the prediction exhibits the visualisation of the experimental behaviour of a SDOF vibration system using the proposed porous bridge bearing, which is how the system behaves in the first resonance. Table 2 shows a comparison of natural frequencies under dry state between the measurement data and the numerical result. From Table 2, it is clear that there is almost no difference in the first resonance frequencies between the numerical and experimental results. This means that in this paper the modal testing used to obtain the dynamic modal parameters of the novel 3DPPBBs is valid. It is important to note that the natural frequency in this section is not equal to that in the tests due to their different boundary condition with/without the plastic box. Since the boundary condition of the bearing system with the plastic box it provides the lower natural frequency when compared to that without the plastic box. This results in a relatively higher frequency due to a stiff floor for its boundary condition.

## 5. Discussion

Table 3 shows the experimental measurements employed for modal parameter identification calculated from Equations (6) and (8). Since the impulse forces of the variation systems occur at the same time, the acceleration and FRFs reduce by more than 10%, 12%, 17%, and 19% at different water levels, showing that compared to the initial condition (dry condition) the speed and size of vibration are considerably slower. Furthermore, when the water flows into the holes of the porous bridge bearing, its natural frequencies can be changed, and resonance frequency slightly shifts initially. However, the big differences of dynamic modal parameters between the dry and complete flood conditions can obviously be observed in Table 3.

It is important to note that in this paper, to enable the modal testing, an idealised single-degree-of-freedom (SDOF) is considered for the proposed bearing’s vibration system due to the extremely lightweight porous bridge bearing specimen. In other words, the specimen can easily move in any direction if there is no large mass on the top of the specimen. Thus it is likely to behave under a free-free condition, resulting in a multiple-degrees-of-freedom (MDOF) system. Thus, the concept method of SDOF is justified to correctly obtain the dynamic modal parameters of the proposed novel 3D-printed porous TPU bridge bearing. These dynamic modal parameters will be useful for bridge engineers to model and develop 3D-printed porous bridge bearings with composite materials under vibration with various environmental conditions. Further work would be conducted on the determination of dynamic modal parameters of these bearings using the principle of a single-degree-of-freedom system shown in this paper to obtain their modal parameters and to comprehend how the bearings will behave under various vibrations with flooding conditions.

## 6. Conclusions

Typical bridge systems have extensively adopted elastomeric bridge bearings, which are one of the most important components in the bridge systems and offer good performance during operations. Their functions are not only to support the weight of a bridge superstructure, but also to accommodate/transfer horizontal and rotational displacements/vertical and horizontal forces to the substructure. However, their performance could be better if using a porous structure with a high performance to weight ratio stated in the introduction section. As a resonance phenomenon can occur in a bridge system under vibration, its dynamic modal parameters of dry conditions are determined in order to safely design and monitor the vibration performance of these bridge bearings before the resonance failure. On the other hand, dynamic modal parameters of a flood porous bridge bearing condition have never been examined before. According to a review in [27], rubber pads in a railway system can considerably face the climate, for example, long-term flooding. Thus, it is required to identify modal paraments of 3D-printed porous bridge bearings (3DPPBBs) subjected to flooding conditions.

In terms of manufacturing 3DPPBBs, they are produced by the fused deposition modeling (FDM) method with thermoplastic polyurethane (TPU) filaments, which have rubber-like properties for bearing conditions. To enable the manufacturing process, the features of a University of Birmingham’s Ultimaker 3D printer are required. Non-destructive field-testing approach involving the impact hammer excitation is considered for investigating the influence of flood conditions on dynamic characteristics of novel 3D-printed porous bridge bearings. Moreover, their vibration system is considered as an idealised single degree of freedom (SDOF), due to their complicated geometry, and extremely lightweight. A range of experimental tests has been conducted under several conditions of flooding levels.

This research is the first to focus on such critical flood conditions. Experiments and numerical analysis are performed to handle such a pressing problem. The experiment results have discovered an unprecedented insight into the dynamic properties of the flooded porous bridge bearings. With the increase in water level, the damping of the bridge bearing system rises, while both the natural frequency and stiffness of the system reduce. It is evident that the dynamic properties of the 3DPPBBs can change considerably under flooding conditions compared to the initial state (dry condition). Unlike flood conditions, the damped natural frequency shifts are relatively small as presented on the FRF curves, the damping increases with the rising water level, but the trend of the system’s stiffness continues to decrease as the natural frequency is proportional to the stiffness. 

In short, this experiment is the world’s first examination of the porous bridge bearing behaviour in flood conditions. The numerical model using these dynamic modal parameters has been established to visualise the experimental vibration behaviour for flooding conditions. Moreover, these insights into modal parameters in flood conditions would offer a new benchmark for the numerical 3D-printed porous bridge bearing modelling in reality. Future study will focus on the determination of dynamic modal parameters of porous bearings with 3D-printed composite materials under various vibrations (referring to a multiple-degree-of-freedom vibration isolation system for bridge bearing applications) with flooding conditions to obtain their parameters used for the numerical modeling of complex porous structures.

## Figures and Tables

**Table 1:** Physical and Mechanical Properties of the Plate and Bearing.

	Thick Steel Plate	Bridge Bearing
Material	Stainless Steel 304	TPU 95A
Mass (kg)	6	0.1
Size (mm^3^)	160 × 100 × 45	160 × 100 × 45
Tensile modulus (MPa)	190	26
Tensile stress at yield (MPa)	205	8.6
Specific gravity	7.9	1.22

**Table 2:** Comparison of first natural frequencies of the proposed bearing under dry condition, being on a ground (without a plastic box) between experimental and numerical results.

Method	Natural Frequency (Hz)	Error (%)
Experiment measurement	65.00	-
Numerical prediction	65.10	0.15

**Table 3:** Dynamic Modal Properties’ Variations under Various Water Levels.

Types of Test	Water Level	Natural Frequency	Damping Ratio	Damped Natural Frequency	Stiffness
	(%)	(Hz)	-	(Hz)	(kN/m)
	0	53.53	0.204	52.40	216.02
Thick Steel Plate	25	51.89	0.204	50.80	203.02
on 3DPPBB	50	51.99	0.204	50.90	203.82
for test 1	75	51.77	0.211	50.60	202.04
	100	51.57	0.212	50.40	200.54
	0	53.40	0.193	52.40	215.04
Thick Steel Plate	25	52.72	0.205	51.60	209.56
on 3DPPBB	50	52.40	0.204	51.30	207.04
for test 2	75	52.17	0.211	51.00	205.25
	100	51.26	0.220	50.00	198.08
	0	53.46	0.198	52.40	215.47
Thick Steel Plate	25	52.82	0.205	51.70	210.37
on 3DPPBB	50	52.27	0.201	51.20	205.97
for test 3	75	52.67	0.210	51.50	209.20
	100	51.64	0.218	50.40	201.08
	0	53.46	0.198	52.40	215.47
Thick Steel Plate	25	52.48	0.205	51.37	207.70
on 3DPPBB	50	52.22	0.203	51.13	205.58
for average test	75	52.21	0.211	51.03	205.49
	100	51.49	0.217	50.27	199.95

## Data Availability

The data that support the findings of this study are available from the corresponding author upon reasonable request.
